# Could nanoparticle corona characterization help for biological consequence prediction?

**DOI:** 10.1186/s12645-014-0007-5

**Published:** 2014-10-01

**Authors:** Emilie Brun, Cécile Sicard – Roselli

**Affiliations:** Laboratoire de Chimie Physique, CNRS UMR8000, Université Paris-Sud, 91405 Orsay, Cedex France

**Keywords:** Gold nanoparticles, Culture medium, Protein corona, Protein identification, Cellular interaction, Hydrodynamic size, Nanoparticle uptake, Nanoparticle toxicity

## Abstract

As soon as they enter a biological medium (cell culture medium for *in vitro*, blood or plasma for *in vivo* studies), nanoparticles, in most cases, see their surface covered by biomolecules, especially proteins. What the cells see is thus not the ideal nanoparticle concocted by chemists, meaning the biomolecular corona could have great biological and physiological repercussions, sometimes masking the expected effects of purposely grafted molecules. In this review, we will mainly focus on gold nanoparticles. In the first part, we will discuss the fate of these particles once in a biological medium, especially in terms of size, and the protein composition of the corona. We will highlight the parameters influencing the quantity and the identity of the adsorbed proteins. In a second part, we will resume the main findings about the influence of a biomolecular corona on cellular uptake, toxicity, biodistribution and targeting ability. To be noticed is the need for standardized experiments and very precise reports of the protocols and methods used in the experimental sections to extract informative data. Given the biological consequences of this corona, we suggest that it should be taken into account in theoretical studies dealing with nanomaterials to better represent the biological environment.

## Background

Since the beginning of the twentieth century [1], manufactured gold nanoparticles (GNP) have been constantly developed for biomedical applications, be it for diagnosis or therapy [2-5]. The enthusiasm aroused by their unique properties, among which spectroscopic and catalytic, and the possible progress they could generate, lead some to talk about a new “Golden Age” [4]. With years, the design of nanoparticles (NP) is complexifying, allowing multiple functionalities on the same object [6-8]. Such sophistication is not achievable with small organic molecules or metallic salts, which accounts for the impetus to consider NP as theragnostic platforms. However, once in a biological environment, NP are submitted to new interactions and constraints that could affect their performance (enzymatic digestion, mechanical stress due to rapid blood flow, corrosion, ligand exchange…). In particular, NP are expected to interact with biomolecules, such as proteins, lipids, nucleic acids and even metabolites, in large extent because of their large surface-to-mass ratio. In fact, the awareness that the synthetic identity of NP could greatly differ from their biological identity is now spreading. Because of proteins omnipresence in biological fluids and the increased number of highly sensitive analytical techniques, there has been a growing number of papers dealing with the formation of a protein corona at the surface of NP [9-12]. Usually, one distinguishes two components in this dynamic process: the soft and the hard coronas. Soft and hard coronas can be defined by their relative affinity for NP surface and exchange times. Hard corona is made by a protein fraction strongly bound to the surface while soft corona is formed by loosely-bound proteins, maybe via protein-protein interaction [10]. The protein corona can thus be multi-layered. NP surface may then get modified and the corona may substantially influence the biological response.

In this review, we will focus mainly on gold nanoparticles (GNP). In a first part, corona characterization will be summarized. We will attempt to draw the main findings regarding what happens to GNP in a biological fluid in terms of size, charge, aggregation state and corona composition. In a second part, we will wonder how this biomolecular corona influences cellular uptake, toxicity, biodistribution and targeting ability.

## Review

### GNP size is expanded by biomolecular corona

**Figure 1:**
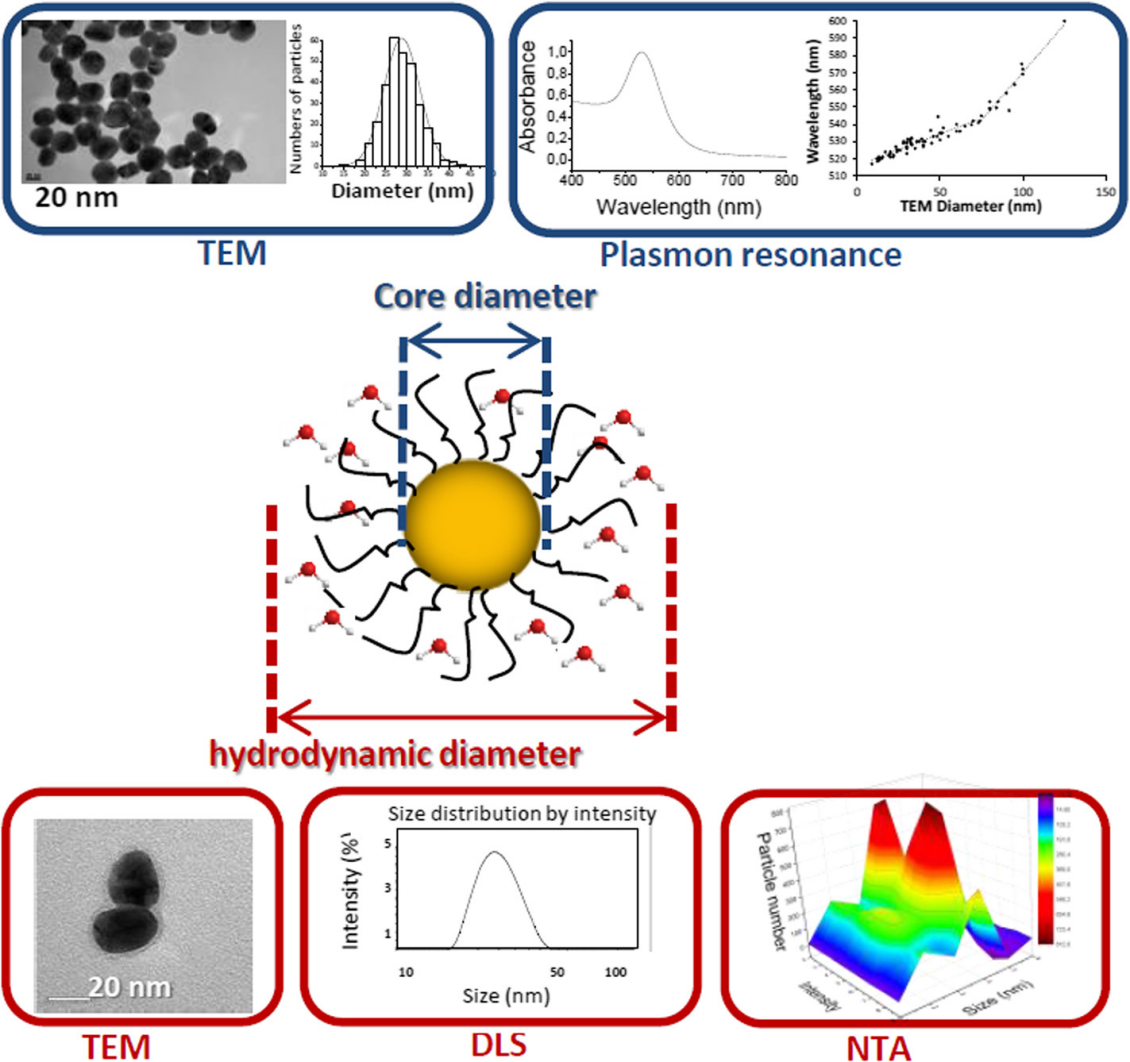
**Schematic illustration of some techniques allowing the determination of NP size.** Such techniques can be classified into two categories, those giving access to the size of the metallic core and those providing NP hydrodynamic diameters. Central part: Scheme of a functionalized NP with the water molecules of its solvation layer. Upper part: TEM and UV–vis spectroscopy allows the determination of the core size. TEM necessitates the measurement of a sufficiently high number of objects to obtain a meaningful distribution as the one presented. Correlation between PR position and NP diameters can be extracted from literature and so UV–vis spectroscopy can give an estimation of NP size in a routine control procedure. Some data were extracted from supplier websites (Sigma-Aldrich and Nanopartz). Lower part: TEM, with an extrinsic coloration, allows the visualization of the biomolecular corona and the measurement of its thickness. DLS and NTA utilize the properties of Brownian motion to provide hydrodynamic diameters. However, the average and distribution given by DLS is weighted by particule size whereas NTA is a particle by particle analysis.

In the attempt to determine metallic NP size, a wide variety of techniques are now available allowing fine characterization [13]. The most commonly encountered are transmission electron microscopy (TEM), absorption UV-Visible spectroscopy to probe plasmon resonance (PR) and dynamic light scattering (DLS). Differential centrifugal sedimentation (DCS) and, more recently, nanoparticle tracking analysis (NTA) were also developed to determine NP hydrodynamic parameters. Figure 1 presents some of the results one can obtain from such techniques. TEM can be considered as the most direct method to visualize NP and determine metallic core shape and dimensions. For reliable results, it necessitates a manual measurement of objects, considered sufficient for a few hundreds of particles [14,15] (Figure 1, left upper part). In addition to core, coloration treatments, with uranyl acetate for example [16], could allow coating visualization and thickness estimation (Figure 1, left bottom part). Specific to metallic NP, plasmon resonance (PR), which is the collective oscillation of electrons at the metallic surface, also gives access to size information [17,18]. GNP plasmon resonance is located in the visible region at *ca.* 530 nm and for citrate-capped GNP, the wavelength of PR varies linearly with diameters from 10 to 70 nm and with a steeper dependence beyond [19-22] (Figure 1, right upper part). This easy-to-use spectroscopy then appears as very powerful and therefore essential for NP size control. Resonance plasmon wavelength is also sensitive to ligand grafting or NP aggregation. Nevertheless, no precise characteristic about the coating can be extracted from such a parameter. On the contrary, DLS [23,24] and NTA [25,26] rely on brownian movements of nanoparticles. Furthermore, DCS [27-29] offers measurements of NP size including both their core and shell according to sedimentation through a density gradient, that allows computing of diameter details linked to the ligand shell in the case of monolayer protected clusters and other particles. Hydrodynamic diameter is defined as the NP diameter implemented with a diffuse layer made of solvent molecules and ions present in solution and with grafted molecules when functionalization was performed (Figure 1, central part). One advantage of DLS is that, contrary to TEM, it allows the analysis of a high number of objects as a few microliters of solution can be scanned by the laser. This statistic analysis leads to an average and accurate size distribution if experimental conditions are carefully optimized [24,30,31]. DLS is more documented for protein corona analysis, especially for GNP, though care should be taken about large particle contribution because the scattered light intensity varies as D^6^ (D = NP diameter), which is not the case for NTA which analyses each particle individually (Figure 1, bottom part). As DLS can provide results in intensity, volume and number, attention should be paid as the raw data are expressed in intensity and do not represent the relative proportions of small and large particles in the sample. To be noticed, some claims that only the hard corona is probed by DLS measurements [32]. As for DCS, one of its advantages is the sensitivity of this method to small surfaces changes [27]. Combination of these techniques then leads to a precise corona thickness depiction.

Table 1 lists GNP characterization in different media. According to these studies, when GNP are dispersed in biological fluids, DLS shows an increase of their hydrodynamic size. As mentioned in several references of Table 1, based on DLS only, agglomeration cannot be excluded. To get information on this point, several authors performed UV-visible spectroscopy and plasmon resonance generally confirms that, in the presence of fetal calf serum (FCS), size increases are the result of protein adsorption: PR shifts due to a change of refractive index. DCS can also give valuable information on aggregation patterns, however it is less used until now. On the contrary, physiological conditions in the absence of FCS seem to induce predominantly agglomeration [33]. Corona formation is expected to depend on several parameters such as size, charge and coating of GNP. For non-coated GNP, according to Maiorano [34], citrate-GNP size determined by DLS in DMEM supplemented with FCS is about 200 nm whatever their original size is (Table 1). This is not in agreement with Wang who showed that 20 nm citrate- NP diameter increases from 20 to 83 nm in DMEM with FCS [35] and with Casals who reported a modification from 24 to 45 nm [36]. For functionalized GNP, this dynamic process, mostly governed by electrostatic interaction, is influenced by the presence of a specific charge and/or coating. In DMEM with serum, COOH-coated objects which exhibit additional negative charge from the carboxylate groups at physiological pH swell up to *ca.* 100 nm whatever their original size [33]. A similar tendency was evidenced by Casals *et al.* but with a lower final size [36]. NP-TTPPBS (bis-sulfonatetriphenylphosphine) in DMEM exhibit a large increase for small objects (<20 nm) and a weaker one for 88 nm NP [32]. It seems anyhow that the smaller GNP, the higher diameter rise. Moreover, the type of medium induces a different behavior as shown by Maiorano comparing DMEM and RPMI for which final sizes and protein adsorption kinetics prove to be different [34].

**Table 1:** **Hydrodynamic diameters of gold nanoparticles : evolution in several biological media**

**NP coating**	**Zeta potential (mV)**	**Diameter (nm)**		**DLS temperature measurement (°C)**	**Hydrodynamic diameter in biological media (nm)**			**NP incubation time in medium**	**Ref.**
		**Core (MET)**	**Hydrodynamic (DLS)**		**RPMI + FCS**	**DMEM + FCS**	**Plasma**		
**citrate**		15			40	200			[34]
		40			70	200			[34]
		80			150	200			[34]
	−21*	20				83			[35]
	−38	30	33	25			76	30 min	[40]
	−34	50	55	25			100	30 min	[40]
	−33	4	5.3			6.1		48 h	[36]
	−44	10	10.1			16.4		48 h	[36]
	−42	13	13.1			22.3		48 h	[36]
	−43	24	24.2			44.7		48 h	[36]
	−45	40	40.6			59.6		48 h	[36]
**NH**_**3**_^**+**^	64	10	12			40.7		48 h	[36]
**COO** ^**−**^		10	24			108			[33]
		25	41			95			[33]
		50	65			88			[33]
		100	97			110			[33]
	−56	10	12			11.8		48 h	[36]
**TPPBS**	−35	5	11	25		63.2			[32]
	−32	15	19	25		75.6			[32]
	−43	80	88.3	25		122.3			[32]
**peptide**	−18*	20				45			[35]

When available, characteristics of nanoparticles are indicated (zeta potential, core diameter, hydrodynamic diameter in water) as well as the media composition and the incubation time. As underlined in the text, most studies do not give details about their DLS measurement conditions.

* = 10%FBS.

These examples highlight the absence of any consensus concerning the extent of GNP size increase by the biomolecular corona. Indeed, it should be noticed that in Wang *et al.*, DLS measurements were performed after centrifugation and resuspension of the sample in a buffer, preparation that could remove a high quantity of loosely bound proteins. DLS temperature measurements should also be considered as it can modify protein/NP association [37]. These particularities illustrate the fact that experimental conditions are decisive. Comparison of different assays can be hazardous when DLS measurements are not performed under identical conditions. Pitfalls could then arise from diversified NP time of incubation in the medium and with cells, temperature of DLS analysis, order of component mixing (serum/medium/NP), …. Then a fine reading and comparison of experimental conditions appear necessary to extract any tendency of NP behavior in physiological medium.

### Corona composition: proteins identification and quantification

Functionalization appears here as a decisive parameter for the quantity and identity of proteins implicated in NP corona. As polyethyleneglycol (PEG) is the most frequently grafted polymer on NP to reduce opsonization, it has been most studied. First, it has been clearly demonstrated by Walkey *et al.* that for a constant GNP size, PEG grafting increase leads to a total protein adsorption decrease [38]. Indeed, weak PEG density was shown to reduce the thermodynamic barrier to protein adsorption. In the case of a constant ligand grafting, the size of the NP seems crucial as its lowering enhances total protein adsorption. This was also demonstrated in ref [32]. Dobrovolskaia also showed that increasing PEG molecular weight grafted on GNP diminishes the total amount of adsorbed proteins [39].

To identify proteins in the corona, two main approaches are commonly used: 1D and 2D SDS-PAGE and mass spectrometry. Nevertheless, here also sample preparation and conditioning can perturb the protein corona. As a consequence, mainly hard corona is analyzed showing anyhow more than nearly one hundred different proteins [40-42].

Electrophoresis, a routine technique, has allowed evidencing the quantity of total proteins and their mass repartition in most studies so far. To be noticed is also the development of micro-BCA or Bradford assay as a tool for relative protein quantification in samples but far fewer papers refer to this technique [43]. NP charge and hydrophobicity appear crucial for the identity of proteins bound to NP as electrostatic interactions are often responsible for the hard corona formation. Unsurprisingly, Casals *et al.* evidenced negatively-charged serum proteins adsorbed on positively-charged GNP [36]. Moreover, small NP seem to bind more specifically small proteins: 5–50 kDa proteins represent respectively 15% and 2% of total for GNP which diameters are <10 nm and equal to 80 nm [32]. Coating density is also a key parameter: Walkey *et al.* identified 147 proteins at the surface of pegylated NP and correlated a high PEG density with a smaller range of protein size present [38]. More precisely, 50 to 80 kDa proteins were more abundant on highly-grafted pegylated NP. In addition, Dobrovolskaia showed that NP pegylation doesn’t change the type of plasma protein composition of the corona though it changes the total amount of proteins [39].

Protein identification has benefited from proteomic approaches and increased sensitivity of apparatus. First, it is to be noticed that the composition of the corona is not the reflect of the surrounding medium, which seems to be independent of the NP type [34,42,44]. By mass spectrometry, the major proteins identified in the corona of GNP in complete cell culture medium are albumin, immunoglobulin and fibrinogen or glycoproteins as can be expected from the presence of FCS [33,34,36]. In addition, complement factor C3 was shown to be predominant for ungrafted NP representing ca. 30% (w/w) of total proteins or 5% for a high density PEG functionalization [32,38]. Dobrovolskaia also detected this complement protein on citrate-coated NP without any induction of activation. It is also important to notice the presence of fibrinogen but without any platelet activation [32,40]. A meaningful example of LC-MS/MS analysis performance is the study of Sisco *et al.*, where distinction between bovine proteins from serum and proteins produced by the rat fibroblasts was achieved, pointing out a possible biological role of rat biglycan protein sequestration in the corona of NR [45]. Albanese *et al.* also profited by the analytical power of mass spectrometry to show that cell-secreted proteins progressively replace serum proteins in the protein corona around citrate-coated GNP in a time- and phenotype-dependent manner, underlying protein corona is a dynamic process [46].

Some studies carefully depicted corona composition with more than one hundred proteins identified. Nevertheless it is utopic to believe that complete corona analysis is accomplished. Given the huge number of different proteins in corona, only part of them is identified. It cannot be excluded that some proteins present at a minor level and so not cited in the literature could be responsible for the major biological consequences discussed below. Considering the variety of nanomaterials in nature, size, shape and coating as long as the different sources of proteins and cell lines, it is difficult to draw absolute conclusions. Still, we believe some trends to be trustworthy. We will uppermost examine studies with GNP but given the paucity of data, we will sometimes refer to other NP.

### The presence of the corona reduces non-specific cellular uptake

A first question of interest is: does the biomolecular corona increase NP uptake by cells? At this point we must distinguish non-specific from specific uptake. Specific internalization is regulated by membrane receptors that are only activated by receptor-specific ligands to trigger internalization. Non-specific uptake is a random process without specific biomolecular control by the cell. We will discuss first the non-specific process.

When studying the impact of serum proteins on cellular uptake, it seems clear that the extent of NP internalization depends greatly on the presence of a corona. Comparing DMEM with and without 10% FBS, Wang *et al.* observed a one order of magnitude higher uptake without serum for two different peptide-coated GNP [35]. For oligonucleotide-functionalized GNP, Patel *et al.* reported a 150% increase in uptake in serum-free medium [47]. Similarly, the uptake of FePt NP or quantum dots (QDs) by HeLa cells were greatly reduced by the formation of a corona compared to the bare NP [48,49]. This trend was also reported for A549 cells with silica NP [50] or with carboxylated polystyrene NP, with the highest uptake occurring in serum-free MEM [51], for human macrophages with a 4-fold uptake of polystyrene NP in HBSS than in 10% human serum RPMI [52], and for mouse macrophages [53]. Once covered with similar biomolecular corona, it seems that same-shaped NP behave the same whatever their core composition. More precisely, the total amount of proteins in the corona seems to impact the extent of uptake. For example, GNP of 15, 40 and 80 nm showed all a different behavior in DMEM and RPMI media supplemented with 10% FBS, with a more abundant corona formed in DMEM. Even though HeLa cells exhibit the same growth rate in the two media, a lower uptake of NP was reported in DMEM [34]. More intriguing, serum heat inactivation also seems to influence NP uptake, even for A549 cells that are known to be insensitive to the complement: a correlation was found between more proteins in the hard corona in the case of heat-inactivated serum and a lower uptake [54]. The reason evoked is the reduction of particles cell membrane adhesion when a biomolecular corona is formed [51,55].

Interestingly, a few studies reported that the biomolecular corona could promote specific uptake: for a couple (NP, cell line) showing the folate-receptor involvement, selective uptake was annihilated without serum [56]. In a differentiated macrophage-like cell line (dTHP1), surexpressing the class A scavenger receptor (SR-A) in charge of the recognition of modified proteins and lipoproteins for their subsequent clearance, Yan *et al.* did not observe any change in effective association and internalization with the presence of serum [55]. But they suggested that the SR-A mediated phagocytosis is only active in the presence of the corona through the recognition of unfolded BSA at the surface of the NP. Prapainop *et al.* also addressed the question of a relationship between misfolded proteins in the corona and cellular uptake by macrophages: they grafted an inflammatory metabolite (cholesterol 5,6-secosterol atheronal B) known to affect protein folding on QDs, and reported a measurable QD uptake for concentration of 10 nM whereas atheronal-free QDs were not taken up by cells even at 100 nM [57]. As for Caracciolo *et al.*, they suggested that NP, through the formation of a protein corona, could target specific cells if among the main coating proteins resides one, still functional, which receptor is overexpressed in diseased cells [58].

So non-specific uptake seems to be decreased in the presence of a corona whereas specific uptake seems to be promoted, sometimes at least, by the protein corona, because a misfolding of corona proteins trigger NP uptake by specific cells that otherwise would not have done so or because there is a protein in the corona able to target a specific receptor expressed in the cell line used. All these results highlight how important each cell line specificity is. Beyond the evident biological relevance of these new findings, they could also allow the reconsideration of a whole part of literature as regards inconsistencies in NP uptake studies as incubation conditions, and especially serum presence, seem to play a major role.

### The presence of the corona generally reduces NP toxicity

As a consequence of a lower uptake, the presence of the corona induces a lower toxicity of nanomaterials. This was, for example, emphasized by a complete set of toxicity assays in Maiorano’s comparison of GNP diluted in DMEM and RPMI [34]. Several observables were quantified: mitochondrial activity through WST-8 assay, membrane integrity with LDH release measurement, apoptosis by flow cytometry and DNA fragmentation with Tunel test. Possible interferences between GNP and the different assays were verified, allowing to state with certainty that the smaller corona GNP induce the higher uptake and the higher toxicity. This has also been reported for carbon nanotubes [59], graphene oxide nanosheets [60] or biopolymeric NP in several cell lines [53]. In the case of well-known toxic nanomaterials such as CTAB-coated gold nanorods (NR) or positively-charged polystyrene NP, the biomolecular corona also plays a protective role as regards membrane damage [61,62]. With FBS-coated CTAB NR, within a 24 h time frame, no morphological impairment of the membrane such as blebs or loss of microvilli was observed, suggesting the corona prevents the amphiphilic CTAB from interacting with the phospholipid bilayer. Interestingly, it has been shown recently that the corona remains bound during internalization and trafficking inside the cell [61-63]. This means that the protective effect of the corona could last as long as it is intact and effectively, a delayed toxicity was observed in the previous cited studies, corresponding to the degradation of the corona inside the lysosomes and the re-exposition of the toxic surface [61-63]. Nevertheless, to the best of our knowledge, such study does not exist yet for GNP.

However, toxicity could be triggered, related to endogenous proteins modifications at the NP surface. This could imply a modulation of biological activity, as observed for cathepsins B and L in the presence of GNP [64] possibly leading to an impairment of the cell machinery, a recognition of immunoglobulins or unfolded protein leading to macrophage activation [55,65] and inflammation [66].

It has also been suggested to take advantage of the protein corona to load small molecular therapeutics such as DNA or doxorubicin [67,68] to induce a toxicity to cancerous cells. Corona seems to act as sponge with a higher payload capacity than what is observed with covalent conjugation strategies. Passive release can be tuned by varying the corona composition and a triggered drug release can be achieved by laser excitation at the longitudinal PR of the gold NR. This pioneering work underlines that, as corona formation is unavoidable, a strategy is needed to exploit it.

### The presence of the corona influences biodistribution

If the influence of NP PEGylation on biodistribution is known for years [69-72], the thorough characterization and consequences of a biomolecular corona formed *in vivo* has not been investigated yet. However, of interest are several studies dealing with a pre-coating of the NP with proteins, namely with serum albumin and apolipoprotein E [53,73,74]. Whatever the nature of the NP core, polymeric or metallic, it seems that such a pre-coating increases the blood circulation time and reduces the clearance speed. For example, a 6-fold increase of half-time was reported by Peng for BSA-pre-coated NP compared to “bare” NP [53]. Based upon *in vitro* experiments, the authors proposed as an explanation a weakened opsonization and a reduced phagocytosis. In all these studies, liver stays the main organ of NP accumulation (more than 90% of the injected dose after 19 h [74]). Still, the protein used for pre-coating seems to nuance the amounts of NP in other organs, albumin targeting lungs preferentially, and brain to a lesser extent, compared to apo-E [74]. One could take advantage of this improved retention when pre-coating NP with albumin in cancer therapy as specific factors account for the accumulation of this protein in solid tumors: a decreased level of HSA in cancer patients, inducing a need for albumin digestion to cover the need in amino acids for tumor growth and the presence of two albumin receptors, gp60 in tumor endothelium and SPARC in tumor interstitium [75].

### The presence of the corona impacts targeting ability

When it comes to therapeutic applications, one main advantage of NP is the multifunctional platform they can become: to address them to diseased cells, functionalization of their surfaces with antibodies, aptamers or other biomolecules is usually involved. Using a click chemistry reaction between azide-functionalized surfaces and bicyclononyne-silica NP (BCN-NP) as a model of targeting, Mirshafiee and coll quantified the targeting efficiencies of such NP in the presence of 10 or 100% FBS : they were lowered by 94 and 99% respectively compared to bare BCN-NP [76]. Such a loss of recognition between the ligand and its target was also depicted in cellular experiments. Constructing gold nanoconjugates with a KDEL-labelled peptide, meant to activate a specific transport pathway, and with a random sequence peptide as a control, Wang *et al.* concluded that, in the presence of serum, non-specific mechanisms of uptake were more robust [35]. Similar results were obtained in the study of transferrin (Tf)-functionalized silica NP [77]: with increasing serum concentration, the targeting capacity of Tf-NP was lost, even when a secondary PEG layer was added to control unspecific protein binding. These observations emphasize that the grafting of a functional ligand is not enough to guarantee the recognition by the corresponding receptor. The biomolecular corona seems to act as a “screen”, preventing NP to discriminate the “right” cells. More recently, Dai *et al.* showed that choosing the correct length of PEG chains could allow to re-establish a selective targeting in the presence of serum [78], suggesting strategies to overcome this difficulty.

## Conclusions

In this review, we wondered what happens to GNP once in cell culture medium. In the presence of serum, what the cell sees is a larger object, the smaller particles being more affected. NP charge can also be modified. Quantity and identity of proteins in the corona are affected by NP size and charge but also by functionalization. In any case, the composition of the corona is not the exact reflect of the composition of the biological fluid: there is a selection process. Extracting general conclusions was awkward as examining this limited body of literature evidences the high importance of carrying standardized experiments, knowing techniques limitations and writing well-documented experimental sections to enable cross-comparisons between studies.

We also highlighted that the interest raised by the biomolecular corona would have been limited without the analytical developments of the recent years. Powerful and sensitive techniques are now available to describe and follow protein corona composition *in vitro*. A new challenge is now to follow it *in vivo*. As culture medium presents a protein composition different from biological fluids, data on protein coronas in both cases could perhaps explain why extrapolation from *vitro* to *vivo* experiments is so difficult.

As for biological consequences, biomolecular corona has pros and cons. On the one hand, it could reduce toxicity, promote in some cases specific uptake and nuance biodistribution, on the other hand it could lead to inflammation processes after activation of macrophages if misfolded proteins are recognized and screen targeted molecules grafted on NP surface. Nowadays, one cannot predict the composition of the corona and its biological consequences: further studies are needed to know how to exploit the benefits of such corona *in vivo*. Moreover, now that biomolecular corona prevalence is well-established, it has to diffuse among a larger scientific community. In particular, it should be included in theoretical studies and simulations, for example dealing with heat transfer induced by NP for hyperthermia. Its consequences in imaging should also be investigated as biomolecular corona might affect fluorescence properties of NP or fluorescent molecules grafted at their surface.

## Abbreviations

Apo-E: Apolipoprotein E
BCA: Bicinchoninic acid
BCN-NP: Bicyclononyne-silica nanoparticle
BSA: Bovine serum albumin
CTAB: Cetyl trimethylammonium bromide
DCS: Differential centrifugal sedimentation
DLS: Dynamic light scattering
DMEM: Dulbecco’s modified eagle medium
FBS: Fetal bovine serum
FCS: Fetal calf serum
GNP: Gold nanoparticle
HBSS: Hank’s balanced salt solution
HSA: Human serum albumin
MEM: Modified eagle medium
NP: Nanoparticle
NR: Nanorod
NTA: Nanoparticle tracking analysis
PEG: Polyethyleneglycol
PR: Plasmon resonance
QD: Quantum dot
RPMI: Roswell Park Memorial Institute medium
SDS-PAGE: Sodium dodecyl sulfate polyacrylamide gel electrophoresis
SR-A: Class A scavenger receptor
TEM: Transmission electronic microscopy
Tf: Transferrin
